# Periodical reactivation under the effect of caffeine attenuates fear memory expression in rats

**DOI:** 10.1038/s41598-018-25648-6

**Published:** 2018-05-08

**Authors:** Lizeth K. Pedraza, Rodrigo O. Sierra, Fernanda N. Lotz, Lucas de Oliveira Alvares

**Affiliations:** 1 Laboratório de Neurobiologia da Memória, Universidade Federal do Rio Grande do Sul, Porto Alegre, Brazil; 2 Laboratório de Psicobiologia e Neurocomputação, Biophysics Department, Biosciences Institute, 91.501-970, Universidade Federal do Rio Grande do Sul, Porto Alegre, Brazil; 3 Graduate Program in Neuroscience, Institute of Health Sciences, 90.046-900, Universidade Federal do Rio Grande do Sul, Porto Alegre, Brazil

## Abstract

In the last decade, several studies have shown that fear memories can be attenuated by interfering with reconsolidation. However, most of the pharmacological agents used in preclinical studies cannot be administered to humans. Caffeine is one of the world’s most popular psychoactive drugs and its effects on cognitive and mood states are well documented. Nevertheless, the influence of caffeine administration on fear memory processing is not as clear. We employed contextual fear conditioning in rats and acute caffeine administration under a standard memory reconsolidation protocol or periodical memory reactivation. Additionally, potential rewarding/aversion and anxiety effects induced by caffeine were evaluated by conditioning place preference or open field, respectively. Caffeine administration was able to attenuate weak fear memories in a standard memory reconsolidation protocol; however, periodical memory reactivation under caffeine effect was necessary to attenuate strong and remote memories. Moreover, caffeine promoted conditioned place preference and anxiolytic-like behavior, suggesting that caffeine weakens the initial learning during reactivation through counterconditioning mechanisms. Thus, our study shows that rewarding and anxiolytic effects of caffeine during fear reactivation can change the emotional valence of fear memory. It brings a new promising pharmacological approach based on drugs widely used such as caffeine to treat fear-related disorders.

## Introduction

Exposure to strong aversive experiences can lead to the formation of enduring traumatic memories, which can trigger many debilitating psychiatric disorders including posttraumatic stress disorder (PTSD) and phobias. In recent years, novel therapeutic strategies that target memory reconsolidation have emerged with promising results to attenuate persistent fear memories. This paradigm involves the transient labilization of the memory trace induced by a retrieval/reactivation session that makes the original memory susceptible to pharmacological and behavioral modifications^1–3^. In animal models, this window of opportunity has been explored by systemic and central administration of different drugs such as protein synthesis inhibitors^4^, blockers of the mammalian target of rapamycin^5^, specific antagonists of NMDA and adrenergic receptors^6,7^, among others. Except for some pharmacological agents used in preclinical studies, for instance propranonol^8,9^ and ketamine^10^, most reconsolidation inhibiting agents are not approved for human testing.

Memory labilization and reconsolidation are not general processes and depend on “boundary conditions”^11,12^. This concept has been used to describe parameters that act as limiting factors for memory to undergo reconsolidation^12^. For instance, both high training intensity and old memory traces are less susceptible to attenuation^13,14^. Given the difficulty in targeting memory reconsolidation in some boundary conditions, another strategy to inhibit fear expression is the enhancement of fear extinction^15^. During extinction, the fear conditioned reminders are repeatedly presented in the absence of footshocks, leading to a progressive reduction of fear expression. Currently, there is a significant need to develop novel pharmacological approaches to accelerate clinical interventions based on reconsolidation disruption or extinction enhancement for fear-related disorders.

Caffeine is one of the most popular legal psychoactive drugs in the world. In the U.S alone, approximately 85% of adults consume caffeine^16^, mainly, but not exclusively through coffee consumption. Caffeine is a non-selective antagonist of adenosine receptors. It is thought that the primary behavioral effect of caffeine is caused by the blockage of the A1 and A2A adenosine receptors^17–19^. Also, caffeine interferes with several adenosinergic regulation processes, including intracellular Ca^2+^ release, inhibition of phosphodiesterases (PDEs), and GABA-A receptors neurotransmission^20^. Numerous studies have addressed the effect of caffeine on memory. Nevertheless, conflicting reports reveal that caffeine may enhance or disrupt memory acquisition, consolidation and retrieval across a variety of fear memory tests^21–26^^.^

On the other hand, caffeine promotes place preference or aversion in a dose-dependent way^27^ and increases the extracellular levels of dopamine and glutamate in the Nucleus Accumbens Shell^28^. Moreover, there are several mixed results showing that acute caffeine injection induces either anxiolytic or anxiogenic-like behaviors in a large range of doses^29–32^.

Despite these several evidence showing that caffeine administrations affect contextual fear conditioning, no studies were performed to evaluate the effects of acute caffeine administration on fear memory reconsolidation to date. Here, we explored for the first time the effect of caffeine administration as a pharmacological strategy to attenuate contextual fear memory in rats.

## Material and Methods

### Subjects

Naïve, male and female Wistar rats (270–320 g/3 months) from our breeding colony were used. Animals were housed in plastic cages, 4 per cage, under a 12-h light/dark cycle at a constant temperature of 24 °C, with water and food *ad libitum*. All experiments were conducted in accordance with local and national guidelines for animal care (Federal Law no 11.794/2008), and the project was approved by the Ethics Committee of the Federal University of Rio Grande do Sul, Brazil.

### Drugs

Caffeine was dissolved in 0.9% sterile saline to obtain the final concentration of 20 mg/kg, based on previous studies^21,22^. Caffeine or vehicle (saline) were administered via intraperitoneal injection (i.p.) in a volume of 1 ml/kg. We used i.p. instead of oral administration in order to guarantee a more precise control regarding both the amount of caffeine received per animal and the exact timing each animal received the treatment before the reactivation sessions.

### Behavioral procedure

#### Contextual Fear Conditioning (CFC)

Along with the experiments, animals were transported from the colony to the holding room. After 30-min of habituation, subjects were moved individually to the experimental chamber and then back to their home cages. The conditioning chamber consisted of an illuminated plexiglas box, 25 × 25 cm, with a metallic grid floor. During training, rats were placed in the chamber for 3-min and then received three possible training protocols: weak training (4 footshocks of 0.4 mA/2 s), strong training (4 footshocks of 0.7 mA/2-s) and very strong training (8 footshocks of 1.0 mA/2-s). Every 4 footshocks were separated by a 30-s interval; animals were kept in the conditioning environment for additional 30-s before returning to their homecages. These training protocols were used based on a previous study^33^.

#### Reactivation sessions

Subjects were re-exposed to the training context at different time intervals depending on the experiment performed, without the US (footshocks) for 5-min. Caffeine or vehicle (saline) was administered 30-min before reactivation sessions.

#### Test Session

Animals were tested for 4-min in the training context 24-h or 6 days after the last reactivation, depending on the experiment performed.

#### Conditioned Place Preference (CPP)

The conditioning box consisted of three chambers, two for the conditioning session with same dimensions (24 × 40 × 50 cm), and the other central/start chamber (10 × 40 × 50 cm). Each chamber was adapted with particular contextual cues and floor texture. The floor of the conditioning chambers was divided into 9 equal rectangles or “sectors” in order to evaluate locomotion along with conditioning and test sessions.

Conditioned place preference consisted of three phases: pre-conditioning (day 1), conditioning (days 2–6) and test (day 7). Animals were injected with vehicle or caffeine before each conditioning session and placed in the chamber associated with the treatment (vehicle or caffeine). The pre-conditioning session (15-min) was intended to reduce novelty and determine initial preferences for any of two chambers using the time spent in each compartment. In case of individual pre-conditioning preferences, conditioning took place in the opposite chamber. For the following 5 days conditioning sessions were performed. Conditioning consisted of pairing one chamber with the injection of caffeine and the other one with the injection of vehicle; animals were in the chamber for 25-min. Animals were submitted to two conditioning sessions a day, for 5 days, separated by a 6–8-h interval between sessions, and received alternating injections of vehicle or caffeine depending on the session. Drug paired-chamber and injection order were counterbalanced across the rats. A 15-min test in the absence of vehicle or caffeine administration was conducted 24-h after the last conditioning day.

#### Open Field (OF)

The open field chamber consisted of a 50 cm height, 60 × 40 cm plywood box and a linoleum floor divided into 12 equal rectangles or “sectors.” In addition, floor was divided into two squares, which allowed the definition of central and peripheral areas. During the experiment, a white room light of ~22 lux was equally distributed into the chamber. The behavior was recorded by video tracking and processed offline. Animals were injected with vehicle or caffeine 30-min before open field exposition. During the 5-min test session, crossings between sectors (locomotor activity) and the time spent in the periphery and center of the apparatus were measured.

### Behavioral Scoring

In all fear conditioning experiments, freezing behavior was registered in real time using a stop-watch and by an experienced observer who was blind to the treatments. Freezing was defined as a tense body posture that resulted from a defensive state with the absence of any movements, except those related to breathing^34^.

During Conditioned Place Preference, the time spent in each chamber was measured and their results were expressed using a preference index (caffeine-paired chamber/caffeine-paired chamber + vehicle-paired chamber). Motor performance was evaluated based on the number of crossings through the sectors.

In the open field test, the number of crossings was considered a measure of motor performance, while the time spent in the center or periphery of the field was considered anxiolytic or anxiogenic responses induced by the treatment, respectively.

### Statistical analysis

After checking for normality (Kolmogorov-Smirnov Test) and Homoscedasticity (Levene test), data were analyzed by repeated measures ANOVA followed by Fisher (LSD) *post hoc* test or independent *t*-student test, with significance set at p < 0.05.

## Results

### Caffeine administration attenuates weak fear memories expression under a classical memory reconsolidation protocol

**Figure 1:**
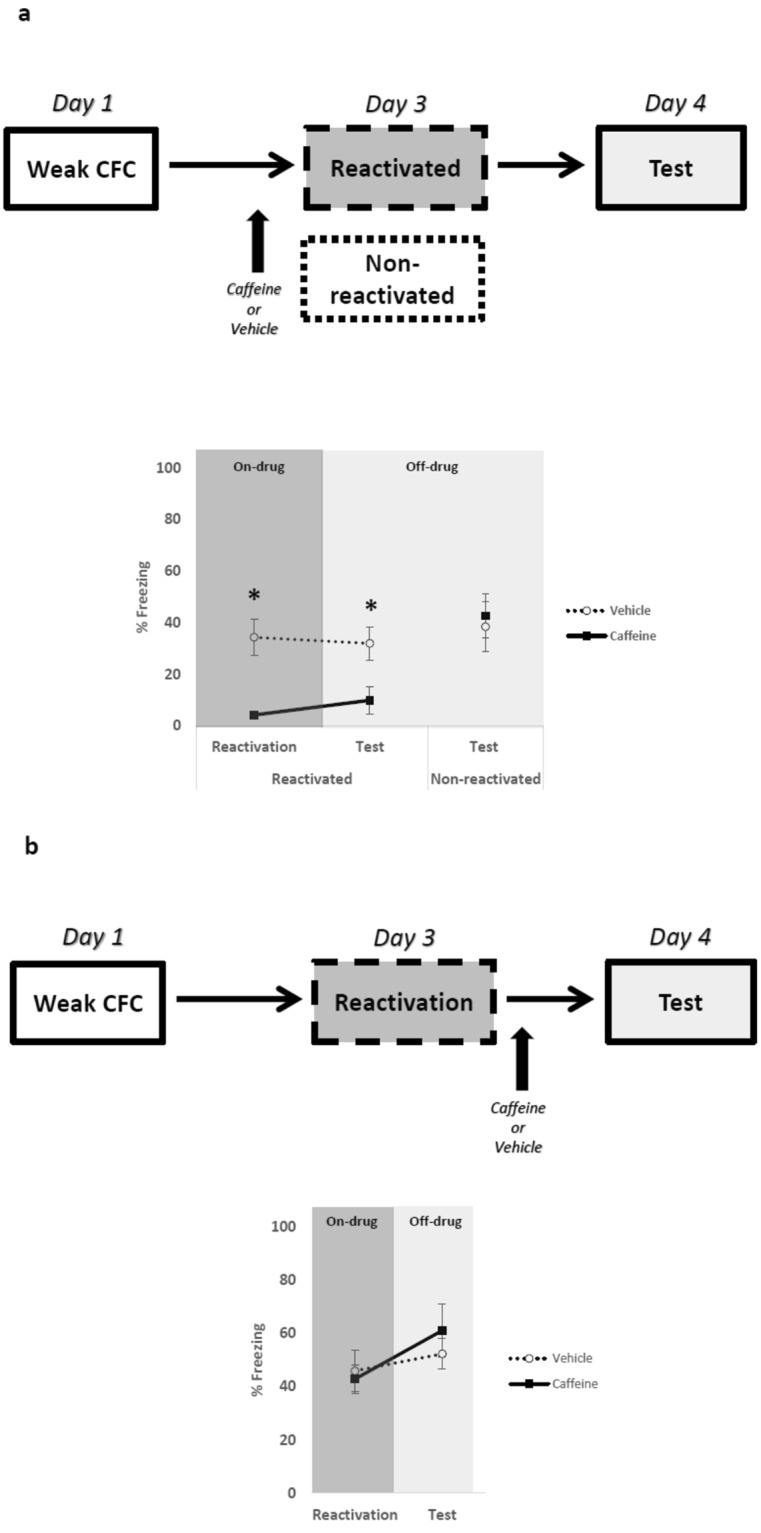
Caffeine administration before but not after single memory reactivation is able to attenuate weak fear memories. The graphs show percent of freezing time expressed as mean ± SEM, and experimental design is shown at the top of each panel; (**a**) Caffeine administration reduces freezing behavior during test with only one memory reactivation after weak training (Vehicle n = 7 and Caffeine n = 6). The same performance was not achieved in non-reactivated animals (Vehicle n = 10 and Caffeine n = 10); (**b**) In addition, there is no effect of caffeine with post-reactivation administration (Vehicle n = 6 and Caffeine n = 7). *p < 0.05, Repeated Measures ANOVA followed by *post hoc* test.

Memory reconsolidation can be affected by pharmacological treatments administered before or after memory reactivation^4,35^. We have previously shown that memory reactivation for 3 to 5-min undergoes memory reconsolidation^2,36,37^. First, we evaluated the effects of systemic caffeine administration before a standard memory reconsolidation protocol. Animals were submitted to weak contextual fear conditioning (CFC) training (4 footshocks of 0.4 mA/2-s) on day 1 and injected systemically with vehicle or caffeine 20 mg/kg, 30-min before memory reactivation on day 3, and tested 24-h later in a drug-free condition. An additional group of animals was submitted to the same protocol; however, they were non-reactivated on day 3. Repeated-measure ANOVA revealed significant effects of treatment factor (vehicle vs. caffeine) (F_1,11_ = 15.077, p = 0.002) but not for time factor (reactivation vs. test) (F_1,11_ = 0.130, p = 0.724) or for treatment x time interaction (F_1,11_ = 0.750, p = 0.404). Post-hoc analysis showed that caffeine-treated animals expressed less freezing during reactivation and test compared to control (p < 0.05). No differences were found in non-reactivated animals (*t* (18) = −0.332; *p* = 0.743, independent *t*-test) (Fig. 1a). This result suggests that standard reconsolidation protocols concomitant with caffeine administration are able to attenuate weak fear memories. Notably, if caffeine was administered immediately after the reactivation session, it did not affect memory expression in the test: Repeated-measure ANOVA revealed significant effects of time factor (reactivation vs. test) (*F*_1,11_ = 6.202, *p* = 0.030) but not for treatment factor (*F*_1,11_ = 0.093, *p* = 0.765) and time x treatment (vehicle vs. caffeine) interaction (*F*_1,11_ = 1.429, *p* = 0.256) (Fig. 1b). This result raised the possibility that endogenous state triggered by caffeine administrations before memory reactivation may be a necessary condition to promote fear attenuation.

### Periodical memory reactivation under caffeine administration attenuates strong fear memory expression

**Figure 2:**
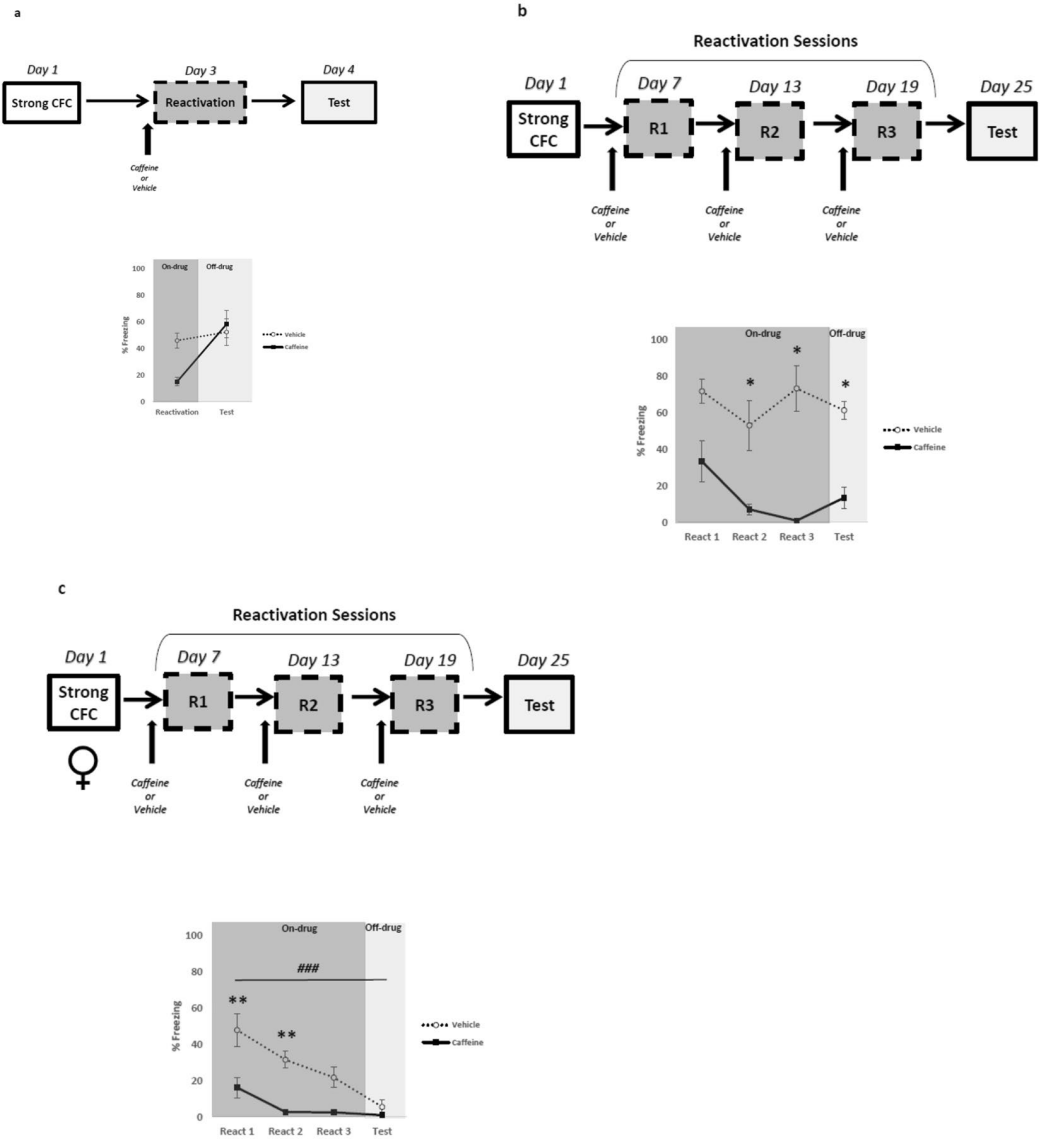
Caffeine administration concomitant with periodical memory reactivation attenuate strong fear memories. The graphs show percent of freezing time expressed as mean ± SEM, and experimental design is shown at the top of each panel; (**a**) The single-reactivation approach fails in strong memories (Vehicle n = 7 and Caffeine n = 7); (**b**) Periodical memory reactivation (3 reactivations, 1 per week) under caffeine administration reduces freezing behavior during the reactivation sessions (on-drug) and test (off-drug) in strong memories (Vehicle n = 6 and Caffeine n = 6); (**c**) Caffeine administration during reactivation reduces freezing behavior in *females (*Vehicle n = 8 and Caffeine n = 9). *p < 0.05, **p < 0.01, ^###^p < 0.001- Repeated Measures ANOVA followed by *post hoc* test. *Differences between groups; ^#^Differences within groups.

It has been shown that memory strength is a critical constraint that limits the effectiveness of fear expression reduction^11,13^. That is, high training intensity induces a boundary condition that prevents reconsolidation interference. In order to evaluate whether caffeine would also be effective at disrupting fear memory reconsolidation in a stronger training condition, animals were fear-conditioned in a higher intensity protocol (4 footshocks of 0.7 mA/2 s). Repeated-measure ANOVA revealed significant effects of time factor (reactivation vs. test) (*F*_1,9_ = 21.856, *p* = 0.001) and time x treatment (vehicle vs. caffeine) interaction (*F*_1,9_ = 12.054, *p* = 0.007) but not for treatment factor (*F*_1,9_ = 1.557, *p* = 0.243). Post-hoc analysis showed that there were no differences between groups during reactivation and test sessions (*p* > 0.05) (Fig. 2a). This result indicated that memory strength is a limiting factor for caffeine efficacy.

Previous experiments of our lab showed that 3-time memory reactivation was able to maintain memory precision^38^ and was effective in modifying the emotional valence of contextual fear memory when associated with rewarding stimuli^5^. Based on these reports, next we evaluated whether additional reactivation sessions associated with caffeine administration would be able to attenuate fear memory in the strong training protocol. Animals were fear-conditioned as described above and were reactivated once a week for 3 weeks. Repeated-measure ANOVA revealed significant effects of treatment factor (vehicle vs. caffeine) (*F*_1,10_ = 21.579, *p* < 0.001) and time factor (reactivation vs. test) (*F*_3,30_ = 8.343, *p* < 0.001) but not for treatment x time interaction (*F*_3,30_ = 0.554, *p* = 0.649). Post-hoc analysis showed that with the exception of reactivation 1 (*p* = 0.082), caffeine-treated animals expressed less freezing in the remaining reactivation sessions (2 and 3) (*p* < 0.05) and during the test session (*p* < 0.05) (Fig. 2b). Taken together, these results showed that additional reactivation sessions under the effect of caffeine disrupted strong contextual fear memory during reactivations (on-caffeine) and test (off-caffeine).

It is well established that males and females respond differently to threat stimuli. For instance, fear-conditioning studies performed in females found less freezing behavior to conditioned stimulus compared to males^39^. Interestingly, sex differences have been associated with individual vulnerability to stress-related disorders such as PTSD^40^. However, most of the fear conditioning studies evaluating the neurobiological mechanism underpinning stress responses have been performed mainly in male rodents. In order to evaluate periodical memory reactivation under the effect of caffeine in females, rats were injected and reactivated 3 times using the strong training protocol, as described above in males. Repeated-measure ANOVA revealed significant effects of treatment factor (vehicle vs. caffeine) (F_1,15_ = 19.691, p < 0.001), time factor (reactivation vs. test) (F_3,45_ = 22.472, p < 0.001), and treatment x time interaction (F_3,45_ = 5.756, p = 0.002). Post-hoc analysis showed that caffeine-treated animals expressed less freezing compared to the control group (p < 0.01) in reactivation sessions 1 and 2. However, these differences were not detected during reactivation 3 and in the test session (p > 0.05). Moreover, there was a significant difference within group in animals injected with vehicle throughout the reactivations and test session (p < 0.05), suggesting an accelerated extinction process in females (Fig. 2c).

### Caffeine administration induces conditioned place preference and anxiolytic response in the open field

**Figure 3:**
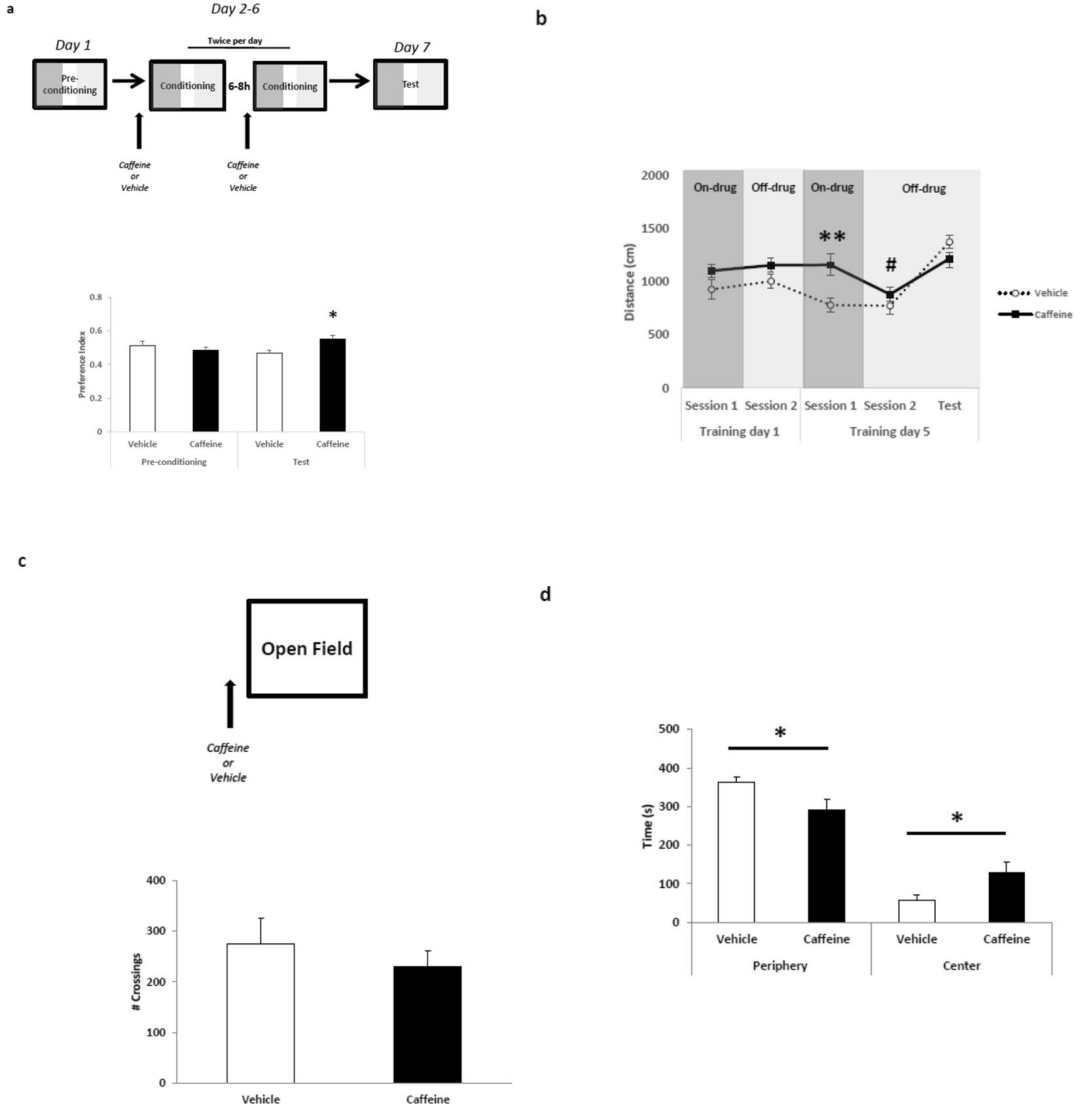
Caffeine induces conditioned place preference and anxiolytic-like behavior in the open field. The graphs show preference index during CPP (caffeine-paired chamber/caffeine-paired chamber + vehicle-paired chamber), number of crossings and time spent in the periphery and center of the open field, respectively. Experimental design is shown at the top of each panel; (**a**) Animals express preference for caffeine-paired chamber compared to control during CPP test session (Vehicle n = 10 and Caffeine n = 8); (**b**) Caffeine-treated animals express higher locomotion during the conditioning day 5 compared to control group and within sessions in an off-drug condition. However, no differences were found on day 1 and in the test session– (Vehicle n = 10 and Caffeine n = 8); (**c**) No difference was detected in number of crossings; (**d**) Caffeine-treated animals spent less time in the periphery and more in the center of the open field, suggesting an anxiolytic effect of acute caffeine administration (Vehicle n = 7 and Caffeine n = 7). *p < 0.05, **p < 0.01 differences between groups (vehicle vs. caffeine) and within sessions (on drug vs. off drug), ^#^p < 0.05, differences between conditioning sessions (day 1 vs. day 5).

It has been shown that fear memory may be reinterpreted to a less aversive level by updating it during reactivation with appetitive stimulus^3^, a pharmacological agent such as morphine^2^, or by preventing freezing expression with an air puff distractor^41^. In order to verify if the effects observed in fear memory disruption are mediated by a possible positive valence stimulus triggered by caffeine during reactivation, we tested its rewarding effect in the conditioned place preference (CPP). Indeed, previous studies have shown that caffeine administration induces place preference in a dose-dependent way^27,42^. In order to address whether the dose used here causes place preference, animals were habituated to CPP and exposed to the conditioning chambers during 5 days (twice per day) with previous administration of vehicle or caffeine. Test session was conducted in a drug-free condition (see Material and Methods for details). Repeated-measure ANOVA revealed significant effects for treatment x time interaction (F_1,16_ = 6.131, p = 0.024) but not for treatment (vehicle vs. caffeine) (F_1,16_ = 1.730, p = 0.206) and time (pre-conditioning vs. test) (F_1,16_ = 0.211, p = 0.652) factors. Post-hoc analysis showed no initial preference during pre-conditioning session between contexts (p > 0.05). Indeed, only 27.7% (5 of 18) of our animals showed initial preferences for one context (upper to 0.55 or lower to 0.45 index preference). However, during test, the caffeine group significantly increased preference for the drug-paired chamber compared to the vehicle group (p < 0.05) (Fig. 3a).

We also evaluated the distance walked during CPP. Repeated-measure ANOVA revealed significant effects for conditioning day (day 1 vs. day 5) (F1,32 = 5.806, p = 0.021), group (vehicle vs. caffeine) (F1,32 = 10.721, p = 0.002) and time (session 1 vs. session 2) x conditioning day interaction (F1,32 = 5.441, p = 0.026), but not for conditioning day and group interaction (F1,32 = 0.420, p = 0.521), time (F1,32 = 0.741, p = 0.395), time x group interaction (F1,32 = 2.766, p = 0.106) and time x day x group interaction (F1,32 = 1.921, p = 0.175). Post-hoc analysis showed that caffeine-treated animals only expressed higher locomotion during the conditioning day 5 (only in session 1) compared to control group (p < 0.01) (Fig. 3b). No differences between the groups were found in the motor activity on the conditioning day 1 (p > 0.05) and in the test session (*t* (16) = 1.573; *p* = 0.135, independent *t*-test). The control group decrease locomotion along the days (p < 0.05). The same pattern was verified in caffeine-treated animals only in free-drug sessions. Taken together, these results indicate that CPP exposition induces context habituation and reduces its locomotion. However, this effect was not shown under caffeine effect. Importantly, in drug-free conditions such as the test session, the caffeine treated group did not show any motor effect.

It has been shown that caffeine induces either anxiolitic or anxiogenic behavior^29–32^. In order to evaluate deeply a possible caffeine effect in motor activity and in anxiety-like behavior, we next submitted animals to an open field test. Animals were treated with caffeine or saline and, 20-min later, exposed in an open field arena. No differences were detected in number of crossings during open field exposition between the groups (*t* (12) = 2.000; *p* = 0.068, independent *t*-test) (Fig. 3c). However, the caffeine treated group spent less time in the periphery and more in the center of the open field compared to vehicle (*t* (12) = 2.371; *p* = 0.035, independent *t*-test) (Fig. 3d).

Our results showed that caffeine in the dose of 20 mg/kg was able to establish conditioned place preference and decreased anxiety responses. The rewarding and anxiolytic profile showed by our treatment allows us to hypothesize that caffeine changes the endogenous state during reactivation, weakening unwanted fear memories through the incorporation of reinforcing information. This “re-signification” phenomenon has been previously shown using natural appetitive stimuli^3^ and opiate drugs^2^ concomitant with memory reactivation.

### Periodical memory reactivation under caffeine administration attenuates remote fear memories

It has been reported that some boundary conditions prevent attenuation of fear memories such as training intensity and memory age. High training intensity makes memory less prone to be affected by pharmacological agents^11,43^. Also, older memories are less susceptible to modification than recent ones^14^. Indeed, long-intervals between acquisition and extinction produce a smaller rate of fear attenuation in rodents^44^ and a more intense hyperarousal compared to early interventions^45^.

**Figure 4:**
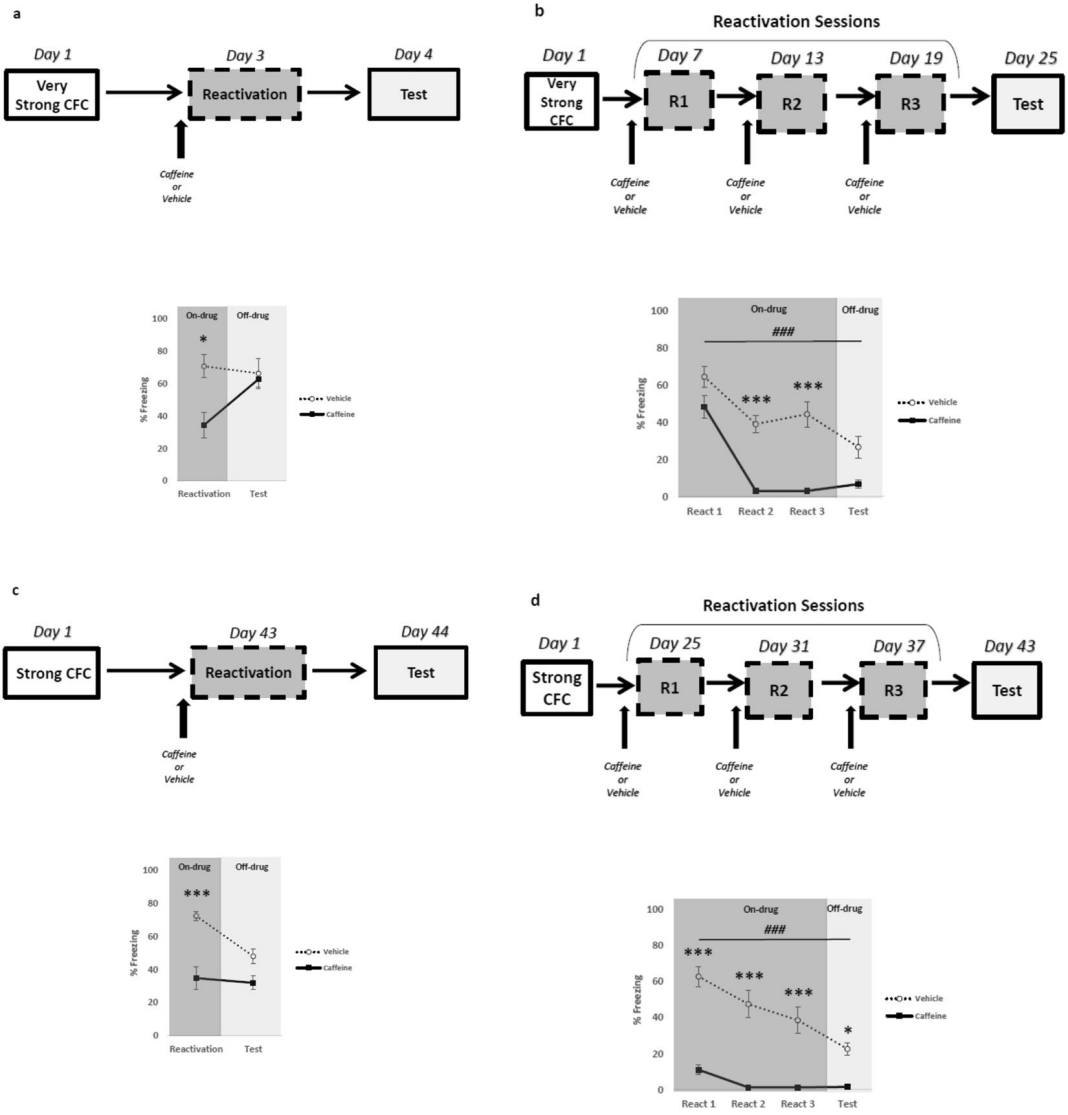
Caffeine administration concomitant with periodical memory reactivation attenuates remote memories. The graph shows percent of freezing time expressed as mean ± SEM, and experimental design is shown at the top of each panel. (**a**) Caffeine administration before a single memory reactivation reduces fear expression during reactivation, but fails to attenuate very strong fear memories (8 × 1.0 mA) in the test (Vehicle n = 6 and Caffeine n = 7); (**b**) Similar results were obtained with very strong memories and periodical memory reactivation under the effect of caffeine (Vehicle n = 9 and Caffeine n = 9); (**c**) Caffeine hinders remote memory during reactivation, but the effect was not persistent during test with a single-reactivation session (Vehicle n = 8 and Caffeine n = 8); (**d**) Periodical memory reactivation under the effect of caffeine reduces freezing expression during reactivation (on-drug) and test (off-drug) of remote memories (Vehicle n = 8 and Caffeine n = 9). *p < 0.05, ***p < 0.001, ^###^p < 0.001, Repeated Measures ANOVA followed by *post hoc* test. *Differences between groups; ^#^Differences within groups.

The following set of experiments were addressed to evaluate if caffeine administration concomitant with memory reactivation would be able to attenuate either very strong training or remote memory. First, animals were trained with 8 footshocks of 1.0 mA/2-s on day 1 and submitted to memory reactivation for 5-min on day 3 with previous administration of vehicle or caffeine. On day 4, animals were tested in a drug-free condition. Repeated-measure ANOVA revealed significant effects of treatment factor (vehicle vs. caffeine) (*F*_1,11_ = 5.004, *p* = 0.04), time factor (reactivation vs. test) (*F*_1,11_ = 5.621, *p* = 0.03) and time x treatment interaction (*F*_1,11_ = 10.559, *p* = 0.007). Post-hoc analysis showed that caffeine-treated animals expressed less freezing during reactivation compared to vehicle (*p* < 0.05). However, no differences were detected during test (Fig. 4a). This result indicated that even after very strong fear conditioning, caffeine may disrupt memory retrieval during reactivation, but this effect is not persistent.

In the next experiment, periodical memory reactivation under caffeine administration was performed in animals trained with 8 footshocks of 1.0 mA/2-s. Repeated-measure ANOVA revealed significant effects of treatment factor (vehicle vs. caffeine) (F_1,16_ = 33.388, p < 0.001) time factor (reactivation vs. test) (F_3,48_ = 51.436, p < 0.001) and treatment x time interaction (F_3,48_ = 5.754, p = 0.001). Post-hoc analysis showed that with the exception of reactivation 1 (p > 0.05), caffeine-treated animals expressed less freezing in the remaining reactivation sessions (2 and 3) (p < 0.05). However, no differences were detected during test (p = 0.058). Moreover, there is a significant freezing decrease throughout the reactivation sessions in vehicle-treated animals (p < 0.05) (Fig. 4b).

Next, we evaluated whether caffeine would affect remote fear memory. When remote memory was evaluated in animals trained with the strong protocol (4 footshocks of 0.7 mA/2-s) and reactivated 43 days later, repeated-measure ANOVA revealed significant effects of treatment factor (vehicle vs. caffeine) (F_1,16_ = 16.248, p < 0.001), time factor (reactivation vs. test) (F_1,16_ = 4.920, p = 0.040) and time x treatment interaction (F_1,16_ = 7.943, p = 0.010). Post-hoc analysis showed that caffeine-treated animals expressed less freezing during reactivation compared to vehicle (p < 0.001). However, no differences were detected during test (Fig. 4c).

Finally, animals were trained with the same strong protocol, but they were reactivated three times (starting on day 25) and tested on day 43. Repeated-measure ANOVA revealed significant effects of treatment factor (vehicle vs. caffeine) (F_1,15_ = 62.746, p < 0.001), time factor (reactivation vs. test) (F_3,45_ = 26.529, p < 0.001) and time x treatment interaction (F_3,45_ = 11.009, p < 0.001). Post-hoc analysis showed that for all reactivation sessions caffeine-treated animals expressed less freezing compared to the control group (p < 0.001). This effect was maintained in the test session in the absence of caffeine (p < 0.05) (Fig. 4d). This result shows that even remote fear memories may be weakened by caffeine administration during reactivation.

## Discussion

In the present study, we evaluated the effect of systemic administration of caffeine concomitant with standard memory reconsolidation protocol and periodical memory reactivation as a potential strategy to attenuate contextual fear memories. Caffeine administration was effective to attenuate weak fear memories through a standard reconsolidation protocol (Fig. 1a). However, the same approach failed when animals were submitted to a strong training (Fig. 2a). A significant fear reduction during test in a drug-free condition was reached in strong memories after 3 periodical reactivations (1 per week for 3 weeks) in both recent and remote memory (Figs 2b and 4d). The dose of caffeine used here was able to establish conditioned place preference (Fig. 3a) and induced anxiolytic effects in the open field (Fig. 3d).

The effects of caffeine consumption on cognition and mood states are well documented. However, there are several reports showing that caffeine effects depend on drug concentration, time consumption (acute vs. chronic), memory phase in rodents (memory acquisition, consolidation, or retrieval) and memory task^21–23,46^. For instance, pre-test injection of caffeine (3 or 10 mg/kg) enhances memory retrieval^24^. However, Corodimas and colleagues have shown that systemic caffeine injection (30 mg/kg) before testing impairs fear memory retrieval^21^. This effect was not state-dependent since caffeine disrupted memory retrieval independent of previous history of caffeine treatment. Accordantly, we have shown here a robust reduction of freezing pattern using 20 mg/kg in the reactivation sessions (under effect of caffeine during retrieval).

The inability to retrieve fear memory in a conditioned context may affect the subsequent fear expression in the same context. We have previously shown that fear conditioned animals that were unable to freeze due to an air puff distractor during the reactivation session expressed low freezing levels in the following tests (in the absence of the distractor)^41^. Thus, one possible explanation for our results is that the retrieval deficits under the effect of caffeine observed during the reactivation may cause a memory change that affects subsequent tests (in a drug-free condition).

Few evidence of caffeine’s reinforcing properties in non-human animals have been shown. Some studies were able to demonstrate preference for caffeine consumption^47,48^ and the enhancement of reinforcement effects of other drugs such as alcohol^49^ and cocaine^50^. Furthermore, Brianna *et al*., showed that acute doses of 6.25, 12.5, or 25 but not 50 mg/kg of caffeine increased the number of active lever presses in an operant conditioning training under progressive ratio schedule for sucrose intake^51^. Also, a low dose of caffeine (3.0 mg/kg) was able to establish CPP while a higher dose (30 mg/kg) produced place and taste aversion^27^. Altogether, these results suggest that caffeine in appropriate doses can be considered a reinforcement enhancer as well as reward stimuli *per se*. In agreement with this view, our results have shown that caffeine induces contextual place preference. In addition, we found that animals under the effect of caffeine spend more time in the central part of an open field arena and less in the periphery compared with the control group, suggesting that this dose of caffeine promotes an anxiolitic-like effect. Indeed, it has been shown that similar doses of caffeine used in the present study induce an anxiety reduction in the open field and elevated plus maze task^29,30^. However, other studies have shown the opposite effect^31,32^. Interestingly, 10 mg/kg in rats correspond to approximately 200 mg of caffeine in human (~2 cups of coffee), a dose that has been associated with positive subjective effects^52^ and memory consolidation enhancement in humans^53^.

The fear reduction shown in our experiments following memory reactivation under the effect of caffeine may be mediated by counterconditioning. This process involves pairing the original conditioned stimuli with a new unconditioned stimuli that has an opposite valence to the original learning^54,55^ (in our case the aversive fear-conditioned context with the rewarding and/or anxiolytic properties of caffeine). Indeed, counterconditioning models applied to PTSD emphasize that negative affective states such as fear or anxiety can be changed by introducing positive stimulus such as pleasurable emotions during exposition^56,57^. Classically, counterconditioning has been considered a reciprocal inhibitory system between aversive and appetitive states that compete for behavioral outcome during retrieval^58^. However, counterconditioning has important differences compared with extinction, since it is based on an incompatible response using stimuli of opposite valence instead of a simple new learning caused by the absence of the US^59^. Despite the fact that counterconditioning has been considered a potential strategy to update memory traces during reconsolidation^60,61^, we cannot rule out the possibility that caffeine accelerates fear extinction since many studies have shown that fear extinction may be enhanced pharmacologically, even in short reactivation trials^62–64^.

In fact, fear reduction over time was verified in control animals exposed to periodical memory reactivation protocol suggesting fear extinction. While time to single exposure in the training context seem to be critical to induce reconsolidation or extinction^65^, repetitive contextual exposition promotes fear extinction^66^. We have previously shown that 3-to-5 min exposure to the context induces memory reactivation/reconsolidation^2,36,37,67^. Accordingly, this allows us to hypothesize that caffeine administration using single memory reactivation may attenuate fear memories via reconsolidation-driven mechanism. However, this assumption cannot be generalized to multiple contextual exposure, which might be acting as an extinction enhancer. Notably, the hypothetical counterconditioning proposed above has been shown during extinction as well as reconsolidation protocols^61,68^, suggesting that memory reactivation concomitant with appetitive valence stimulus is a general mechanism to promote fear attenuation. Because predictability of exposure to conditioned stimuli^69^ and differences between massed and long-spaced exposures^44^ are able to influence the extinction outcome, future experiments should be conducted to address the effects of caffeine under these conditions.

The requirement of repetitive memory reactivation coupled with caffeine administration is in accordance with a time-dependent process necessary for the effective incorporation of an endogenous state induced by the treatment. Interestingly, similar results were reported by our group with the consumption of appetitive stimuli during reactivations^3^. In this study, the presence of chocolate during three reactivation sessions of fear conditioned animals resulted in a context-appetitive stimulus association that changed the memory emotional valence to a less aversive level, weakening fear expression in the following tests (even in the absence of chocolate). This suggests that when a contextual fear memory is reactivated in the presence of an appetitive stimulus (chocolate or caffeine), such positive information may be incorporated into a preexisting memory, retuning its emotional valence to a less aversive state.

It has been shown that a remote memory is less likely to be modified or updated than a recent one^70,71^, although there are divergent results^11,72^. In fact, remote memories seem to be resistant to pharmacological disruption of reconsolidation^13,14^ and induce fear incubation^73^. Although some studies have been shown enhanced fear reduction in early vs. late interventions^44,45,69^, we found that caffeine was able to attenuate fear memory even in long intervals between training and test. Nevertheless, it remains to be investigated if additional responses such as fear generalization and hyperarousal could also be attenuated.

In females, although caffeine induced a freezing reduction during reactivation, there was no effect in the test. Probably this result was caused by the low freezing levels observed in the control group. In the animals conditioned with the very strong training protocol, caffeine was able to reduce freezing expression in the reactivation sessions, but only a tendency in the test. Indeed, this discrepancy between fear expression during memory reactivation and test have been thoroughly demonstrated, showing that the performance during the within-session does not necessarily predict the between session fear expression^69^. It is possible that caffeine may be effective even in a very strong condition if some parameters are adjusted, such as the number of reactivation sessions or its duration. Importantly, even with small differences in freezing behavior across training protocols, we showed that the conditioning strength as well as reactivation length may be critical factors to determine the caffeine effect in memory. Thus, it is possible that the behavioral expression may not correspond strictly to cellular and molecular mechanisms underpinning memory processes^11,74^.

In the last decade, several studies have shown that fear memories can be attenuated by interfering with the reconsolidation process with different drugs^4,6,7^. However, most of these pharmacological agents cannot be administered to humans. Our findings show that fear memory can be weakened using a widely used drug during memory reactivation. Nonetheless, the fact that caffeine intake in humans are commonly chronic in society might be a limitation to translate our findings for humans and requires further studies. Preclinical and clinical research on caffeine effects on memory reconsolidation and extinction could bring new therapeutic approaches for fear-related disorders such as PTSD and phobias.
