# Author Correction: Periodical reactivation under the effect of caffeine attenuates fear memory expression in rats

**DOI:** 10.1038/s41598-018-28934-5

**Published:** 2018-08-06

**Authors:** Lizeth K. Pedraza, Rodrigo O. Sierra, Fernanda N. Lotz, Lucas De Oliveira Alvares

**Affiliations:** 1 Laboratório de Neurobiologia da Memória, Universidade Federal do Rio Grande do Sul, Porto Alegre, Brazil; 2 Laboratório de Psicobiologia e Neurocomputação, Biophysics Department, Biosciences Institute, 91.501-970, Universidade Federal do Rio Grande do Sul, Porto Alegre, Brazil; 3 Graduate Program in Neuroscience, Institute of Health Sciences, 90.046-900, Universidade Federal do Rio Grande do Sul, Porto Alegre, Brazil

Correction to: *Scientific Reports* 10.1038/s41598-018-25648-6, published online 08 May 2018

In the original version of this Article, the author Lucas De Oliveira Alvares was incorrectly indexed. This error has now been corrected.

The version of this Article previously published contained lower resolution images for Figures 1, 2, 3 and 4. This has now been corrected in the PDF and HTML versions of the paper.

